# Supplementary material for: Are carbon emissions trading and green financial instruments synergistic? -Comprehensive quantitative research based on content analysis
Source: PLoS One. 2024 Mar 7;19(3):e0298601. doi: 10.1371/journal.pone.0298601 (PMC10919601; doi:10.1371/journal.pone.0298601)
Supplement: S1 Data — (ZIP) [file pone.0298601.s001.zip › Data/地级市Z_F工作报告.zip!/╦─┤¿/╥╦▒÷╩╨/└┤╫╘╣1⁄2╓┌║┼í╛┬φ┐╦╩2╛▌═°í┐_2017.docx]

各位代表：

我代表市人民政府，向大会报告工作，请予审查，并请市政协委员提出意见。

过去五年工作回顾

过去五年，是宜宾发展进程中极不平凡、极富挑战的五年。在省委、省政府和市委的坚强领导下，在市人大及其常委会的法律监督、工作监督和市政协的民主监督下，我们紧紧依靠全市人民，认真履职尽责，圆满完成本届政府各项目标任务，为决战决胜全面小康奠定了坚实的基础。

过去五年，我们攻坚克难、加快发展，综合实力进一步增强。面对持续复杂的宏观经济形势和经济下行压力，我们主动适应和把握经济新常态，全力稳增长、调结构、促转型，全市经济保持平稳较快发展、产业转型迈出坚实步伐。预计全市地区生产总值从2011年的1091.18亿元增加到2016年的1650亿元，年均增长9.3%；人均GDP从24433元增加到36700元，年均增长9.2%；规上工业增加值年均增长10.3%；一般公共预算收入从67.23亿元增加到125.3亿元，年均增长13.3%；社会消费品零售总额从397.72亿元增加到762.5亿元，年均增长13.9%。民营经济占地区生产总值比重达56.8%。预计三次产业结构由15:61.9:23.1调整为14：57.5：28.5。工业转型步伐加快，白酒等传统支柱产业稳步回升，页岩气等高端成长型产业正在形成。净增规上工业企业175户、总数达到665户，工业总产值突破2150亿元、利润居全省第2位。13个重点产业园区累计建成面积48平方公里，入驻企业744户。临港开发区列入长江经济带国家级转型升级示范开发区，五粮液园区列入国家级新型工业化示范基地，四川长江工业园区正式启动并加快建设。农业农村经济稳步发展，茶叶、肉牛等特色产业加快壮大，全市农林牧渔业总产值预计达到410亿元，年均增长4%。服务业加快发展，预计实现增加值468亿元，年均增长10.6%。宜宾港集装箱吞吐量从2011年的8055标箱增加到2016年的30.1万标箱，年均增长106%。引进金融机构16家，金融业增加值年均增长18.5%。旅游收入年均增长25%。连续三届蝉联国家科技进步先进市，国家知识产权试点城市通过验收，成功创建全国质量强市示范城市。

过去五年，我们抢抓机遇、力推项目，发展后劲进一步提升。抢抓中央和省上持续加大项目投入的历史性机遇，牢牢把握项目投资对经济增长的关键作用，全力推进重大项目建设。预计累计完成固定资产投资5576亿元，年均增长19.3%。产业支撑项目建设成效明显，向家坝水电站、中电福溪电厂、华电珙县电厂、天原新材料产业园、金川电子西部磁电产业园等项目建成投运，江之源智能终端产业园、腾卓智能终端制造基地等项目开工建设。交通项目建设取得新进展，宜泸高速、宜叙高速建成通车，成贵铁路、宜宾机场迁建、宜彝高速、绕城高速西段等加快建设，渝昆铁路、川南城际铁路（蓉昆高铁）宜宾段、宜西铁路、成宜高速、宜攀高速等项目前期工作有序推进。水利项目建设进展顺利，王家沟、新坝等中型水库加快建设，向家坝灌区北总干渠一期和长江上游干流四川段防洪堤工程前期工作有序推进。谋划储备未来五年重大项目800个、投资规模超过10000亿元。

过去五年，我们做大城市、做实县域，城乡统筹进一步加快。城市总体规划、历史文化名城保护规划自撤地设市以来首次通过省政府批准，编制实施组团控规及专项规划80个。预计城镇化率从2011年的39.5%提高到47.1%，年均提高1.5个百分点。城市建设全面推进，中心城区建成区面积108平方公里、常住人口108万人，建成“双百”大城市。长江公园、高速公路出入口改造、旧城立面整治、城市道路黑化等重大市政工程基本完成，长江生态综合治理项目快速推进。县域经济加快发展，预计GDP超过100亿元的县（区）由2011年的2个增加到8个。小城镇建设取得新成效，入选全国重点镇12个、全省“百镇建设行动”试点镇18个。新农村建设成效显著，累计建成省（市）级新农村示范县（片）13个、幸福美丽新村905个。

过去五年，我们深化改革、扩大开放，发展空间进一步拓展。深化行政审批制度改革，取消市级行政审批事项和公共服务事项106项、非行政许可审批事项30项。户籍制度改革实现“四个全面放开”。我市被列为9个国家电子招标投标试点省市之一。农村改革深入推进，我市被确定为全省唯一整市推进新型林权抵押贷款改革试点市。大力实施创新驱动，累计创建高新技术企业50户、创新型企业76户、国家级产业化基地1个，获国家、省级科技立项260项，申请专利6883件。对外开放持续深化，与国内外13个城市结为友好城市。宜宾港保税物流中心（B型）、进境粮食指定口岸建成运营。累计引进项目2418个，到位资金3664.49亿元，丹麦嘉士伯、阿里巴巴等一大批国内外知名企业入驻我市。累计实现外贸进出口总额42.86亿美元。外事、侨务、台事、贸促、海关、检验检疫等工作取得新成绩。

过去五年，我们倾力惠民、促进和谐，人民生活进一步改善。累计拨付民生工程专项资金492.69亿元，各项民生工程、民生实事全面完成。预计城镇和农村居民人均可支配收入分别达到28360元、12860元，年均分别增长10.4%、11.8%。累计投入扶贫资金130.5亿元，贫困人口减少41.8万人，贫困村退出75个，贫困发生率下降到3.82%。累计转移输出农村劳动力800余万人次，新增城镇就业32.5万人、城镇登记失业率控制在4%以内。实施农村义务教育“四大工程”项目2624个，完成投资30.5亿元。宜宾卫校、宜宾市职业技术学校完成整体搬迁。2016年高考本科上线人数首次突破1.5万人大关。市广电中心、市档案馆新馆等大型文化设施建成投用。环境质量持续改善，污染物减排任务全面完成。成功创建为中国杰出绿色生态城市和四川省环境保护模范城市、森林城市、园林城市，生态县（区）创建名列全省第一。“法治宜宾”“平安宜宾”“健康宜宾”建设全面深化。安全生产形势总体平稳，食品药品监管力度持续加大。国防后备力量持续加强，连续四届荣获全国双拥模范城称号，国防动员、国防教育、兵役征集、交通战备、人防、司法行政、审计、监察、消防、气象、应急管理、信访维稳、防灾减灾等工作扎实推进，民族宗教、新闻出版、电子政务、地方志、爱国卫生、住房公积金、红十字、科协、档案、保密、工会、青少年、妇女儿童、老龄、残疾人等事业全面发展。

过去五年，我们改进作风、提升效能，发展环境进一步优化。认真落实中央八项规定和省委省政府、市委市政府十项规定，深入开展党的群众路线教育实践活动、“三严三实”专题教育、“两学一做”学习教育，积极推进环境兴市战略和“双千”活动，制定实施转作风、抓落实、促发展等规定，发展氛围日益浓厚。着力推进依法行政，获得第三届“中国法治政府奖提名奖”和“2015年度中国政府创新优秀实践奖”。实施“五个公开”，全力推进阳光政府、高效政府建设。政务大厅行政审批当日办结率85%，按时办结率100%。强力正风肃纪，惩治和预防腐败体系建设扎实推进。

过去五年，我们在发展实践中积累了宝贵经验，也为今后一段时期提供了重要启示。一是必须坚持专注发展不动摇。只有坚持不懈调结构、转方式、促升级，聚精会神打基础、补短板、增后劲，加快走出“追兵渐近、标兵渐远”的境地，才能推动全市高位求进、跨越赶超。二是必须坚持改革开放不动摇。只有坚定不移地推进各项改革，主动融入大局、发挥优势，打造好于沿海、优于全省的发展环境，加快引进大项目、大企业、大集团，形成大产业，才能不断激活发展内生动力。三是必须坚持生态优先不动摇。只有尊重自然、顺应自然、保护自然，坚持绿色发展，切实担负区位、生态赋予我市的重任，加快构建长江上游生态屏障，才能实现“既要绿水青山又要金山银山”。四是必须坚持改善民生不动摇。只有把改善民生作为一切工作的出发点和落脚点，积极回应群众关切，真正为老百姓解难事、做实事、办好事，才能不断提升全市人民的获得感和幸福感。五是必须坚持务实落实不动摇。只有坚持在市委的坚强领导下，求真务实、勇于担当，尽心尽力抓工作，不折不扣抓落实，才能形成一心一意谋发展、干事创业争一流的强大合力。这些经验是五年来发展实践的高度升华，值得我们好好坚持和发扬。

各位代表！以上成绩和经验的取得，是省委、省政府和市委坚强领导的结果，是市人大及其常委会和市政协监督支持的结果，是全市各族人民和各级各部门共同努力的结果。在此，我代表市人民政府，向各位人大代表、政协委员，并通过你们向全市各族人民、驻宜部队、武警官兵、政法干警、各民主党派、工商联、无党派人士、各人民团体和各界朋友，表示衷心的感谢，并致以崇高的敬意！

成绩值得铭记，发展永无止境。我们清醒地看到，在全面建成小康进程中依然面临不少困难。传统产业转型升级压力较大，新兴产业发展不足，新的增长动能尚显薄弱；城镇化相对滞后，旧城提升、棚户区改造任务较重；县域经济发展不平衡，部分县（区）园区产业空心化；脱贫攻坚、民生改善任务艰巨，优质公共服务资源供给不足。我们将全力以赴解决这些问题，决不辜负人民重托！

未来五年主要任务

今后五年，是宜宾创新发展、转型升级的加速期，脱贫攻坚、全面建成小康社会的决胜期。新的时代、新的起点，赋予我们新的任务、新的使命。我们将继续抢抓国家西部大开发、长江经济带、“一带一路”、成渝经济区建设和省委省政府加快推进川南经济区一体化发展等重大机遇，始终保持发展定力、战略定力，坚定信心、奋勇争先，奋力谱写全市经济社会发展新篇章。

今后五年的基本思路和奋斗目标是：高举中国特色社会主义伟大旗帜，以马克思列宁主义、毛泽东思想、邓小平理论、“三个代表”重要思想、科学发展观为指导，深入贯彻落实习近平总书记系列重要讲话精神和治国理政新理念、新思想、新战略，紧紧围绕“五位一体”总体布局和“四个全面”战略布局，坚定不移践行中央“五大发展理念”和省委“三大发展战略”，全面落实市第五次党代会的战略部署，以绿色发展、科学发展、加快发展为取向，以供给侧结构性改革为主线，以全面创新、开放合作为路径，加快建设经济强市、区域中心大城市、区域交通枢纽城市、全面创新改革先行市和幸福美丽城市，确保全面建成小康社会。

今后五年重点办好十大实事：

1.坚决打赢脱贫攻坚战。确保2019年全市农村贫困人口全部脱贫、贫困村全部退出和贫困县全部“摘帽”。

2.产业转型取得决定性成果。加快推进传统产业转型，培育形成千亿级企业1户、三百亿级企业2户、百亿级企业3户、十亿级企业50户。全力培育新兴产业，加快发展轨道交通、智能终端、新能源汽车、通用航空、新材料、节能环保、医药及器械、页岩气等高端成长型产业和绿色食品加工业，形成百亿级产业6个，新兴产业工业总产值达2000亿元以上。大力推进特色农业产业化，茶叶、肉牛、生猪综合产值分别达到300亿元、500亿元、500亿元。

3.城市建设实现质的飞跃。投资1000亿元以上，完成绕城高速、机场迁建、长江生态综合治理、三江口开发、天原片区改造建设、纸厂片区改造建设、南广立交、盐坪坝长江大桥、南部新区开发、成宜高速、岷江五粮液湿地公园（暂定名）、城市生态修复（荒山裸坡专项治理）等项目，形成区域中心大城市的核心支撑。

4.构建区域交通枢纽。投资1000亿元以上，打造铁、公、水、空立体交通体系，形成宜宾至成都、重庆、昆明、贵阳的“1小时交通圈”“两小时交通圈”，基本实现“县县通高速、县县通铁路”。

5.建设旅游强市。大力发展全域旅游，蜀南竹海、兴文石海、五粮液景区、李庄古镇创建国家5A级旅游景区，到2021年旅游总收入达到1000亿元，游客接待量1亿人次。

6.打造现代科教强市。加快宜宾大学城、科创城建设步伐。实现更高水平的义务教育均衡发展，基本普及高中阶段教育、学前3年教育，实施中等职业教育铸品牌提品质工程，促进民办教育市场化、特色化、品牌化发展，推进智慧教育全覆盖，培育一批名校、名师、名校长。

7.完善城乡居民社保和医疗体系。稳步提高城乡居民社保待遇。完成“三基一卡”信息化建设。从2017年起开展以全民免费体检为主要内容的全民预防保健工作，惠及全市7—64岁人群约355万人。全市三级公立综合医院达到5个、三级公立中医医院达到2个。

8.建成一批百姓安居工程。投资215亿元，改造棚户区40518户。改造农村危房8.5万户。

9.实施一批重大水利工程。计划水利总投资88亿元以上。完成长江上游干流四川段防洪堤工程，基本完成向家坝灌区北总干渠一期工程，建成王家沟、新坝、东山、龙滚滩、二龙滩、蟠龙湖、仁和等7个中型水库。

10.筑牢长江上游生态屏障。深入开展大规模绿化宜宾行动，新增绿化造林面积150万亩。打好环境污染防治“三大战役”，实现城镇和重点乡村生活污水、垃圾处理设施全覆盖，切实改善环境质量。成功创建国家卫生城市、国家园林城市、国家森林城市。

今后五年，我们将突出抓好六个方面的工作：

一、突出发展为要、产业为本，建成经济强市

建设经济强市，根本在发展。我们将坚持巩固提升传统产业和加快发展新兴产业“双轮驱动”，促进一二三产业融合发展，建成总量大、结构优、质量好、贡献大、绿色佳的经济强市，成为川渝滇黔结合部区域经济中心“领头羊”，到2021年全市GDP达2500亿元左右。

全力做大做强传统优势产业。加快白酒、能源、化工、轻纺、建材等现有支柱产业改造升级，推动现有支柱产业精细化、高端化发展，产业增加值年均增长9%以上。巩固提升以“五粮液”为龙头的“宜宾酒”品牌形象，着力整合白酒品牌、延伸白酒产业链、壮大产业集群，白酒主营业务收入在全国、全省同行业中的占比分别达到20%、45%；协调推进电力、新能源和核燃料等能源产业发展，建设四川综合能源深度开发基地；改造提升粘胶纤维、氯碱化工、精细化工等优势产业，重点培育醋酸纤维素和新型节能建材等具有区域竞争力的产业。大力推动建筑业转型升级和加快发展，进一步实施建筑业大企业倍增计划，到2021年建筑业总产值达到600亿元以上。加快构建茶、牛、猪、竹、蚕现代特色优势产业体系，培育壮大农业产业化龙头企业，到2021年全市农林牧渔业总产值达到550亿元，形成百亿级农业产业5—6个。加大旅游景区开发打造力度，完善道路、酒店、游客接待中心等旅游服务功能，加快推进旅游资源大市向旅游强市转变。完善物流基础设施建设，大力发展以港口物流、工业物流、商贸物流等为重点的多式联运，到2021年实现物流产业增加值200亿元。加强金融服务与产品创新，引进或新设金融机构10家以上，到2021年实现金融产业增加值130亿元。积极发展家庭服务、批发零售、住宿餐饮等服务业。

全力培育壮大新兴产业。组建200亿元规模的新兴产业投资公司，大力发展八大高端成长型产业和绿色食品加工业，到2021年，新兴产业增加值占规上工业增加值的比重达50%以上。大力发展轨道交通产业，全力打造集核心技术研发、整车总装、零部件生产、运营维保、综合服务为一体的轨道交通产业基地，到2021年实现总产值220亿元。加快发展智能装备产业，打造智能终端产业集聚区，到2021年实现总产值550亿元。着力发展新能源汽车产业，加强电池、电机、电控等核心部件创新研发和项目引进，到2021年实现总产值550亿元。科学规划布局通用航空制造业，重点发展以通用航空器组装、无人机研发制造、通航材料及关键零部件为主的通航制造业，到2021年实现总产值200亿元。大力推进页岩气产能建设、开发和就地转化利用，加快形成新能源产业链，到2021年形成产能45亿立方米/年，建成全国重要的页岩气生产基地。加强新材料、节能环保、医药及器械等产业发展，引进一批旗舰企业、链核企业、骨干企业。大力发展绿色食品加工业，加快构建一批绿色食品加工业全产业链，到2021年实现总产值700亿元。

全力加快县域经济发展。建设经济强市，基础在县域、重点在县域、潜力在县域、难点也在县域。我们将始终把县域经济发展摆在突出位置，放心、放手、放活、放大、放强县域经济，到2021年GDP突破200亿元的县（区）达到4个。按照因地制宜、分类指导的原则，加强县域产业发展研究和谋划，每个县培育2—3个支柱产业。研究制定县域经济发展的支持政策，落实好我市扶持工业企业发展用电用气补贴办法等政策措施，推动沿江经济区和山区资源综合开发区协调发展。加大民营经济的政策扶持力度，引导和扩大民间投资，民营经济增加值年均增长8.7%以上，到2021年民营经济占全市经济总量的比重达58%。实施园区发展层次提升工程，完善管理体制、运行机制及运营模式，支持符合条件的园区创建国家级、省级高新技术产业园区和开发区，到2021年园区入驻企业达到1000户，园区产值占全市工业总产值的85%以上。

全力构建内陆沿江开放高地。按照“东进西融、南联北接”的思路，全方位提升开放合作层次和水平，全市招商引资市外到位资金年均增长8.5%左右。主动对接世界500强、全国500强和成长性好的企业，加快引进一批大项目、大企业、大集团，不断提高产业招商的比重。积极争取国家、省上重大工业项目布局和产业政策支持，建立健全区域产业合作机制。依托黄金水道、交通大通道等资源，大力发展临港经济、通道经济，主动融入长江经济带建设。全力打造一流发展环境，对支撑作用强、科技含量高、产业链条长的重点产业，从政策扶持、要素保障等方面给予重点支持。着力提升开放合作实效，积极参加万企出国门、中外知名企业四川行、西博会等重大经贸投资促进活动，办好中国（宜宾）白酒文化节、国际（宜宾）茶业年会等重大投资促进活动和展会。鼓励和支持本地在外企业家情感回报、资金回流、项目回投，引导在外发展的企业家、技术专家返乡创业。鼓励有条件的企业到省外甚至国外开展研发设计、品牌收购、兼并重组等，培育本土跨国公司。

二、突出品质为要、以人为本，建成区域中心大城市

当前，宜宾正处在新型城镇化加速期、机遇期、转型期，我们将着力做大城市规模、提升城市功能，加快建设规模适度、品质一流、环境优美、功能完善、辐射吸纳能力强的区域中心大城市，到2021年中心城区建成区面积超过160平方公里、常住人口超过160万人。

切实创新城市发展理念。宜宾拥有“三江六岸”的自然生态资源、2000多年的历史文化传承、优势明显的区位交通条件、特色鲜明的产业支柱、553万各族人民的共同愿望、多年城市建设形成的发展基础，已经完全具备建设区域中心大城市的条件和支撑。我们将牢固树立“不求最大、但求更佳”的城市发展理念，按照一流的规划、一流的特色、一流的人居环境，认真处理好高起点规划与高标准建设的关系、有山有水与显山露水的关系、文化传承与形象展示的关系、城市建设与产业发展的关系，大力彰显城市特色和品味，提升城市发展精气神。牢固树立“质量至上、精益求精”的城市建设理念，按照一流的建设、一流的管理，在设计、施工和管理上精雕细琢、精打细磨，打造一批骨干工程、支撑工程、精品工程。牢固树立“以城建城、以城兴城”的城市经营理念，按照一流的体制机制，着力破解制约城市发展的征地拆迁、资金筹措、建设模式等问题，推动城市建设迈上良性发展轨道。

切实提升城市建设品质。做好“山、水、园、林、路、景”文章，把整体风貌设计、色彩基调、建筑单体形态等全程融入城市规划建设，做到水在城中、城在园中、楼在林中、人在绿中，让城市与人、自然、环境和谐融合。大力实施城市生态建设工程，科学划定城市绿线，逐步提升城市自然生态，到2021年中心城区累计建成城市公园73个、街旁绿地283.79公顷、滨河景观绿带189.77公顷，城市绿地率达35.4%。充分挖掘和弘扬宜宾特色文化，注重在开发中保护、在保护中开发，培育城市文化元素、文化气质，对外树立形象，对内凝聚人心。加快新型智慧城市建设，推动各领域信息化建设与应用，提高城市现代化、信息化、智能化水平。大力推进海绵城市建设，有序推进地下综合管廊建设。实行严格的城市管理，进一步理顺城市管理体制机制，规范行政执法，切实构建市容整洁、交通通畅、运转有序的城市环境。

切实加快中心城区建设。加快推进中心城区“两横两纵”骨干道路建设，打通组团与组团间的连接通道，完成城市核心框架建设。全力推进南部新区（含金沙新区）、岷江新区、临港新区、南溪新区建设，拓展城市发展空间。加大城市修补力度，推进城市亮化、净化、美化工程，进一步疏解老城人口、完善城市功能，促进老城区建筑物、街道立面、城市绿化和色彩环境更加协调、更加优美。加快棚户区、危旧房和老旧小区改造，重点推进天原片区、纸厂片区、三江口等区域改造提升，打造城市发展亮点。

切实提升辐射吸纳能力。制定出台与建成区域中心大城市相匹配的政策体系，培育壮大现代商贸、教育服务、文化、医疗、会展等产业，提升区域生产要素配置、信息资源交流、技能人才培训等方面的承载能力，让宜宾充满活力、吸引力、凝聚力。着力壮大现代商贸业，加快推进三江口中央商务区建设，提升城市商业层次。着力实施城镇教育资源倍增工程，培育一批名校、名师，积极发展优质民办教育，探索发展混合制教育，发展具有区域比较优势的高品质学校。加快构建现代公共文化服务体系，大力推进演艺娱乐、出版印刷、动漫创意等文化产业发展。着力用好现有医疗资源，加快弥补中医、医药方面的短板，打造区域一流的医疗服务中心。加快推进城际干道、城镇道路和公交一体化建设，建设有轨电车示范线。加强城市垃圾、污水处理和停车场、洗车场、农贸市场等配套设施建设，不断完善城市功能。

切实提高户籍人口城镇化率。突出以人的城镇化为核心，深化农业转移人口市民化相关配套改革，在就业创业、子女入学、社会保障、医疗服务等方面实现同城同待遇，让他们进得来、留得住、过得好。加快县城扩容提质，引导人口、产业向县城集聚，力争打造7个10万人口以上的小城市。推动“产、城、人、文”融合，打造特色小镇，培育提升一批专业镇、示范镇，带动农村人口就地就近转移。

三、突出功能为要、互通为本，建成区域交通枢纽城市

坚持大交通引领大发展，通过“拓展骨架、提升功能、完善路网”，加强交通基础设施建设，完善互联互通连接线，实现铁路、公路、水运、航空“四港”互动，构建快捷、安全、高效、覆盖城乡的综合交通运输体系，到2021年铁路通车里程达到600公里、高速公路突破600公里。

加快建设铁路港。着力构建连接川滇、川黔、攀西的“四高七普一枢纽”铁路体系，成为全国重要地区铁路枢纽和四川省铁路枢纽次中心。抢抓宜宾列入全国50个铁路枢纽之一、国家“八纵八横”高速铁路网中“京昆”“兰广”两大重要通道在宜宾交汇的重大机遇，加快成贵铁路、川南城际铁路（蓉昆高铁）宜宾段、渝昆铁路、隆黄铁路、宜宾至西昌铁路、珙县至叙永铁路、盐津至筠连铁路等重大项目建设。

加快建设公路港。着力构建“一绕九射”12条高速公路网，建成绕城高速、宜彝高速、成宜高速、宜威高速、仁沐新高速，加快宜攀高速、内南高速等公路建设。加快推进“七横六纵”干线公路网提档升级，建成宜叙高速竹海连接线、宜叙高速僰王山互通至石海景区连接线、南溪至泸州快速通道（宜南阳泸快速通道）、宜庆路等地方干线公路。

加快建设水运港。大力推进宜宾港4个港区11个作业区建设，不断完善港口集疏运体系，到2021年，宜宾港集装箱吞吐能力达100万标箱、吞吐量实现60万标箱，建成四川最大内河港和长江上游绿色生态港。实现长江航道Ⅲ级升Ⅱ级、金沙江和岷江航道升Ⅲ级，长江航道实现2000吨级以上船舶常年昼夜通航。

加快建设航空港。建成宜宾新机场，开辟更多的航线，积极争取开通国际航班，旅客年吞吐量达100万人次，货邮吞吐量3800吨。设立开放口岸，大力发展空港物流产业和空港经济。依托菜坝机场，全力打造集研发、制造、运营、观光、教育为一体的通用航空小镇。

四、突出引领为要、科技为本，建成全面创新改革先行市

抢抓四川建设全面创新改革试验区机遇，把全面创新改革作为引领发展的“一号工程”，力争在关键环节创新改革上取得决定性成效，加快培育创新驱动转型发展新动能。

加快建设大学城。积极推动国内外知名高校在宜创办合作大学，引进国内知名高校在宜设立新校区，鼓励社会资本在宜创建高职院校，支持宜宾学院创办应用型综合大学、宜宾职业技术学院建设国内一流职业院校，加快建成四川健康职业学院，到2021年新建成高等院校3—5所，在校大学生人数超过6万人。

加快建设科创城。深化与同济大学、四川大学、电子科技大学、哈尔滨工业大学、西南交通大学、西华大学、四川理工学院等高校合作，加快建设一批产业技术研究院、工程技术研究中心、重点实验室等科技研发机构，到2021年国、省级科技研发机构达到30家。深化科技体制改革，支持和鼓励五粮液、天原、丝丽雅、中核建中等重点企业开展先进技术标准研制，将具有比较优势的企业标准上升为国家标准。完善成果评价激励机制，加快科技成果转化。培育壮大核能和核技术应用产业、军工电子产业等军民融合支柱产业，打造国家级军民融合示范基地。

深化供给侧结构性改革。深入推进“三去一降一补”，着力化解煤炭等行业过剩产能，去除无效供给、扩大有效供给，建立健全与供给侧结构性改革相适应的政策体系和长效机制。深入推进农业供给侧结构性改革，大力推进农业标准化、绿色化、品牌化生产，支持开展“三品一标”创建和申报，增加绿色优质农产品供给。大力振兴实体经济，坚持以提高质量和核心竞争力为中心，加强质量监管和品牌建设，统筹大企业大集团培育和中小微企业创新发展，新增规上工业企业200户以上。合理引导住房消费，建立购租并举的住房制度，促进房地产市场平稳健康发展。

深化重点领域改革。全面完成各项改革任务清单，形成和推广一批经验成果。深化行政管理体制改革，进一步转变政府职能、精简审批事项。深化商事制度改革。深化投融资体制改革，用好用活市国资公司等平台，依法依规加强政府与社会资本合作，包装推出一批打基础、利长远、高含金量的政府与社会资本合作项目。深化国资国企改革，坚持因企施策、分类推进，积极稳妥推进混合所有制改革和股权多元化改革，力争用三年时间完成新一轮市属国有企业改革。深化农业农村改革，落实农村土地集体所有权、农户承包权、土地经营权“三权分置”办法，加快培育新型农业经营主体，积极发展适度规模经营。

全力推进大众创业、万众创新。办好“双创”活动周等重大活动。依托企业、高校、科研院所等科研机构，加快大数据中心、科技孵化器、众创空间等创新创业平台建设。深入实施高层次人才“千人计划”、创新型企业家培育计划、科技创新领军人才和青年科技创新研究团队培养支持计划，引进培育一批经营管理型、专业技术型、科技领军型、创新创业型人才。

五、突出脱贫为要、强农为本，建成全面小康社会

坚持以脱贫攻坚统揽经济社会发展全局，把脱贫攻坚作为未来五年的头等大事和第一民生工程，落实责任，挂图作战，确保按期完成脱贫目标。

坚决打赢脱贫攻坚战。紧扣“两不愁、三保障”和“四个好”要求，认真落实“六个精准”“五个一批”“五个一”等系列部署，强化主体担当，集中力量攻坚。压实脱贫责任，严格执行党政主要领导负总责、党员干部“一岗双责”，落实扶贫目标、任务、资金、权责“四到县”制度。整合脱贫资源，着力统筹金融、社会、政策、人才等资源，形成政府、市场、社会互为支撑的大扶贫格局。创新脱贫模式，因地制宜构建特色产业基地带动、市场主体带动、股权造血增收带动、跨村联建支部带动等脱贫模式，千方百计增加贫困群众收入。健全脱贫机制，采取现场推进、专项督查、行业报告等措施，对工作落实不力的地区和部门实行最严厉的问责。强化脱贫保障，加大财政专项投入力度，实施差别化考核，加强贫困村基层组织建设和群众思想引导，提高贫困主体自我发展能力。

大力培育扶贫产业。精准脱贫关键靠产业。针对不同区域、不同类型、不同致贫原因，因村制宜、因户施策，加快培育贫困地区、贫困群众增收产业体系，到2019年，初步形成“跨乡联县”特色支柱产业体系，确保贫困地区产业助农增收人均增幅高于全市平均水平，贫困县农村居民人均可支配收入比2010 年翻一番以上。用好浙江省嘉兴市对口帮扶屏山县等机制，加强与东部沿海地区的产业协作、精准对接。积极推广“借牛还牛”等资产收益扶贫经验，打造脱贫攻坚宜宾模式。大力发展“互联网+”、电商扶贫等扶贫新形式，着力打造好“百企帮百村”“种子基金”“爱心包裹”“爱心结对帮扶”“雨润计划”等社会扶贫品牌。

大力改善农村生产生活条件。加快推进农村交通、农田水利、电力、能源、通讯等基础设施建设，增强物资集散、公共服务和居住功能，解决贫困地区行路难、饮水难、用电难、通讯难等突出问题。开展易地扶贫搬迁、库区移民、避灾搬迁等10.28万人。大力实施幸福美丽新村建设“五大行动”，新建成省级新农村示范县4个、市级新农村示范县7个、幸福美丽新村2400个，65%以上的行政村建成省级“四好村”，85%以上的行政村建成市级“四好村”。

六、突出民生为要、和谐为本，建成幸福美丽城市

坚持人人参与、人人尽力、人人共享，千方百计保障和改善民生，让改革发展成果更多、更公平、更公正、更实在地惠及广大人民群众。

加大民生投入促进“保障实”。积极实施一批普惠性民生工程，确保全市财政民生投入占公共财政支出比重保持在65%左右。建成覆盖城乡的社会保障体系，完善基本医疗保险制度，做好社会保险关系跨地区转移接续工作。完善社会福利服务体系，健全老年人、未成年人和残疾人等特殊群体关爱服务体系，积极发展慈善事业。城乡居民人均寿命由74.78岁提高到76.1岁。大力实施保障性安居工程，推进棚户区改造和城镇保障性住房建设，货币化安置比例90%以上。

强化就业创业促进“增收快”。大力弘扬“工匠精神”，加大就业培训力度，鼓励和引导广大劳动者提高综合素质和专业技能，打造一批敬业、精业、勤业的专业人才队伍。有针对性地做好返乡农民工、企业下岗人员、大学毕业生、退伍军人、贫困群众等重点人群就业工作，城镇登记失业率控制在4.2%以内。到2021年，城镇居民、农村居民人均可支配收入分别达到42755元、19787元。

完善公共服务促进“生活好”。实施城乡义务教育一体化发展工程，加大农村、边远、贫困和民族地区教育投入，确保所有县（区）通过全国义务教育发展基本均衡县国家评估验收。积极发展普惠性学前教育。实施普通高中软实力提升工程，全面提升普通高中办学水平。大力发展职业教育，深度推进产教融合、校企合作。完善公共文化服务体系，回应群众多样化文化生活需求。大力争创全国文明城市。积极推进“健康宜宾”建设，全面深化卫生计生改革，建立健全医疗卫生服务体系，强化重大疾病预防控制。大力培养医疗专业人才，严格医疗执业标准，推动医疗资源向基层、农村流动。加强食品药品安全监管，让老百姓吃上放心食品、用上放心药。积极发展体育事业和产业，开展全民健身运动，提高竞技体育水平。切实抓好民族宗教、新闻出版、地方志、爱国卫生、红十字、电子政务、科协、档案、工会、青少年、妇女儿童、老龄、残疾人等事业发展。

坚持绿色发展促进“环境美”。加快建设长江上游绿色生态市，以“三江九河”生态功能区为重点，深入推进退耕还林和天然林资源保护工程，实现宜林荒山、荒坡、荒丘、荒滩造林绿化和天然林管护全覆盖，森林覆盖率提高到49.59%。大力发展特色经济林产业，连片建成10个“万亩林亿元钱”示范区。大力改善环境质量，加强重点流域、重点区域、重点行业环境污染整治，到2021年中心城区空气质量优良天数比例提高到80.7%，PM2.5年均浓度比2016年下降10%以上，市、县（区）城市生活污水处理率分别达到95%、85%，乡镇生活污水处理率达到80%。深入推进绿色低碳产业发展，大力发展清洁能源和能源装备产业，建成城市生活垃圾焚烧发电厂，推进煤炭资源清洁高效开发和综合利用，全力打造临港开发区、翠屏区节能环保装备产业基地。大力普及环境知识，倡导绿色办公、绿色生活和休闲模式。

创新社会治理促进“秩序优”。坚持党委领导、政府主导、社会协同、公众参与、法治保障，提高社会治理体系社会化、法治化、智能化、专业化、精细化水平。完善城乡网格化服务体系。积极支持群团组织参与社会治理，规范发展各类社会组织。推进社会治安综合治理，加快治安防控体系建设，持续开展打非治违和重点专项整治，健全公共安全体系。坚决杜绝暴力恐怖案件、极端恶性事件发生。扎实开展安全生产隐患排查，落实自然灾害防范措施，强化以公安消防为骨干的多种形式应急救援力量建设。健全群众诉求表达、利益协调和权益保护机制，按照法定途径分类处置信访问题，及时排查预警和调处化解社会矛盾。

强化国防建设促进“军民融”。强化国防动员和后备力量建设，支持国防和军队改革，巩固和促进军政军民团结。深入开展双拥模范城（县、区）创建活动。切实做好国防动员、国防教育、人民防空、交通战备、优抚安置等工作。

2017年重点工作

各位代表，未来五年奋斗目标已经明确，2017年是落实各项工作的开局之年，我们将坚决贯彻党中央国务院、省委省政府和市委的决策部署，坚持稳中求进、进中求好、好中求快，从高处着眼、从实处着力、从细处着手，推动经济平稳健康发展与社会和谐进步。

今年的主要发展目标是：GDP增长8.5%左右；规上工业增加值增长10%左右；建筑业增加值增长10%左右；服务业增加值增长10%左右；全社会固定资产投资增长13.5%左右；社会消费品零售总额增长13%左右；一般公共预算收入增长8.5%左右；城镇化率提高2个百分点以上；城镇新增就业5.5万人以上，城镇登记失业率控制在4.2%以内；城镇居民人均可支配收入增长8.5%左右；农村居民人均可支配收入增长9.5%左右；完成省上下达的物价调控、节能减排等目标任务。

围绕上述目标任务，我们将着力抓好以下工作。

一、坚定不移坚持“第一要务”，保持经济较快增长

牢牢把握发展第一要务，主动应对风险挑战，推动全市经济平稳较快发展。深化供给侧结构性改革。继续依法依规化解煤炭等行业过剩产能，做好人员安置工作。把房地产去库存和促进人口城镇化结合起来，加大棚改和征地拆迁货币化安置力度，将商品房库存控制在合理范围。规范政府举债行为，积极防范和化解地方政府性债务风险。认真落实各类税费减免政策，全面清理规范涉企收费，降低企业生产经营成本。全力弥补脱贫攻坚和基础设施、公共服务、生态环境等短板。努力扩大有效投资。确保绕城高速、城市生活垃圾焚烧发电、嘉士伯重啤生产线技改等重大项目建成投运，全面完成S436姚家嘴至沙河公路路面改造、筠连至巡司快速通道建设。大力推动成贵铁路、宜宾机场迁建、成宜高速、宜彝高速等重大交通基础设施建设，加快实施宜庆路、宜叙高速竹海连接线、宜叙高速僰王山互通至石海景区连接线、兴文县纳黔高速连接线二期工程、南溪仙源长江大桥以及6个中型水库等重点项目建设，尽快形成实物投资增量。开工建设向家坝灌区北总干渠一期工程、长江上游干流四川段防洪堤工程。启动南溪至泸州快速通道前期工作。全力促进页岩气开发和就地转化利用、中核建中压水堆核燃料生产线搬迁、智能终端制造基地等重大产业项目建设。大力推进市二医院临港医院等民生项目建设。积极扩大市场消费。进一步优化城市大型商业综合体、购物中心、农贸市场等网点结构和布局。大力发展电子商务，推进电子商务进农村、进乡镇、进村社，培育新的消费热点。

二、坚定不移实施“双轮驱动”，促进产业转型升级

着力实施“双轮驱动”，一手抓传统优势产业的巩固提升，一手抓新兴产业的培育壮大，确保完成工业投资418亿元、技改投资320亿元。加快推进五粮液“千亿工程”、丝丽雅和天原“三百亿工程”、中核建中“百亿工程”，鼓励有条件的企业实施跨行业、跨区域兼并重组。继续推进“个转企”“企升规”“规改股”，加快培育新的工业增长点。全面组织实施八大高端成长型产业和绿色食品加工业发展规划，加大项目引进和推介力度，推动具有投资意向的企业尽快签约、落地，加快四川轨道交通产业项目、江之源智能终端产业园、腾卓智能终端制造基地等已开工项目的建设进度，力争全市新兴产业总产值突破200亿元。优化产业园区布局，探索建立园区间招商与产值、税收等利益分享机制，建立健全园区投融资、研发设计等公共服务平台。着力实施“十三五”服务业发展规划和五大新兴先导型服务业专项行动方案，加快临港国际物流园区、象鼻物流园区、菜坝配送中心等物流节点建设，宜宾港集装箱吞吐量增长15%以上。认真落实加快旅游业发展的扶持政策，大力培育旅游消费市场，积极推进蜀南竹海、兴文石海、李庄古镇等景区创建国家5A级景区，五粮液景区成功创建4A级景区并启动5A级景区创建工作，打造一批具有宜宾特色文化的精品景区、线路、演艺体验产品、文化创意商品。培育壮大现代金融业，抓好宜宾市农商银行组建等工作。扎实推进省级现代农业、林业、畜牧业重点县建设，大力培育休闲农业、康养农业、农村电商等新产业新业态，重点打造茶叶、生猪、肉牛、林竹、蚕桑等特色产业。

三、坚定不移抓住“三个关键”，激发发展动力活力

全力推进“创新、改革、开放”三个关键领域的工作，不断提升经济发展层次和水平。着力创新驱动。加快建设大学城，大力引进国内外知名高校入驻，开工建设一批高校和实训实习基地；加快建设科创城，筹建一批产业技术研究院、博士后工作站、工程技术中心、重点实验室等相关研发机构，建成科技孵化器6个、“众创空间”60个。着力改革推动。深化投融资体制改革，大力推动简政放权，全面推进审批、核准、备案权限承接、下放，切实落实企业和基层投资自主权。积极推广应用PPP模式，吸引社会资本参与项目建设运营。统筹城乡综合配套改革，重点推进农村综合性改革、供销社综合改革、农业科技体制改革，继续深化农村产权制度改革。着力开放带动。制定完善招商配套政策，鼓励资金重点投向高新技术产业、现代服务业，引导优秀人才来宜创业、投资。用好各类重大投资促进活动平台，大力推进精准招商，创新中介招商、委托招商、网上招商等方式，招商引资市外到位资金突破900亿元。抓好项目对接落地，迅速掀起比项目、比投资、比落地、比效益的引资热潮。加快实施“走出去”战略，鼓励企业开展境外经贸合作和园区建设，全年外贸进出口总额达8.5亿美元。

四、坚定不移推动“四区同步”，提升城市建管水平

同步推进以南部新区、岷江新区、临港新区、南溪新区等四区为重点的中心城区建设，不断做大城市规模、提升城市品质。加强城市设计、建筑立面规划编制和管理，完成组团控规和专项规划19个。抓好县级行政区划调整。南部新区：建成金沙江大道一期工程，抓紧推动外江路以北、金沙江大道以西的项目，迅速拉开城市骨架，打造城市特色和亮点，形成布局合理、配套齐全、交通便捷、环境优美、生活舒适的新片区。岷江新区：完善城市配套设施，加快推进滨水文化特色街区建设，开工建设岷江五粮液湿地公园，打造交通组织流畅、建筑风格协调、景观特色显著、区域品质高端的江北组团中心。临港新区：按照“产学研港城”融合发展思路，加快建设科教之城、产业之城、开放之城、宜居之城。开工建设盐坪坝长江大桥，加快推进天原片区改造建设等项目。南溪新区：着力完善城市功能，推进宜宾至南溪有轨电车交通项目规划建设，大力发展生态经济、赛事经济、旅游经济、康养经济，进一步聚集人气、商气、财气。同时，加快推进三江口片区拆迁、招商、建设进度，着力打造融时尚、文化、高端为一体的现代“城市客厅”。深入推进城市执法体制改革，健全大城管工作机制，强化城市管理日常巡查、监督、考核。进一步整合信息资源，拓展数字城管在占道停车、城市照明等智能管理方面的应用，做好城市交通组织、园林绿化等工作。

五、坚定不移紧扣“五个一批”，完成年度脱贫目标

按照“五个一批”脱贫攻坚路径，聚焦年度脱贫目标，认真实施“六大兜底”“17个扶贫专项”方案年度计划，确保贫困人口脱贫6.73万人，贫困村退出180个，实施易地扶贫搬迁2.74万人。扶持生产和就业发展一批。用好用活贫困村产业扶贫周转金和扶贫小额信贷，通过“龙头企业+农户”“专业合作社+农户+基地”等形式，力争每个贫困村都有龙头企业和专业大户带动。积极开发公益性岗位，引导与发达地区开展劳务合作，鼓励用人单位吸纳贫困人口就业。移民搬迁安置一批。有序开展易地扶贫搬迁、库区移民、生态移民、避灾搬迁，培育和扶持搬迁贫困群众后续产业发展。低保政策兜底一批。加强政策性扶贫力度，扩大社会保险覆盖面。对丧失劳动能力、无法通过产业扶持和就业帮助实现脱贫的贫困家庭，按政策纳入农村最低生活保障。对农村无劳动能力、无生活来源、无法定赡养扶养义务人或其法定义务人无履行义务能力的特困人员，实行自愿集中供养。医疗救助扶持一批。全面落实“十免四补助”、贫困地区基本医疗卫生服务“8个100%”等政策，实施先诊疗后结算，确保贫困患者县域内住院医疗费用全报销、慢性病门诊维持诊疗个人支付占比控制在10%以内。发挥医疗救助基金的作用，切实解决因病致贫返贫问题。灾后重建帮扶一批。对洪涝、滑坡、泥石流等灾区贫困人口，科学制定灾后重建规划方案，尽快重建家园、重塑产业。

六、坚定不移开展“六项行动”，推进美丽宜宾建设

一是推进生态屏障建设行动。实施大规模绿化宜宾行动，完成年度造林目标任务。成功创建国家森林城市。强力推进生态市、县（区）、乡镇创建，完成高县、筠连县、珙县、兴文县省级生态县创建工作。二是推进大气污染防治行动。实施减少工业大气污染物排放、抑制城市扬尘、压减煤炭消费总量、治理机动车船污染、秸秆禁烧五大工程，提升空气环境质量。三是推进水污染防治行动。全面落实“河长制”，加强“三江九河”水环境污染综合整治，加快各县（区）城市污水处理厂提标改造和二期工程建设，确保杨湾污水处理厂建成投运。加大中小河流治理和新出险病险水库整治力度，加强页岩气开发中的污染监控和治理，综合治理水土流失面积120平方公里。四是实施土壤污染防治行动。开展土壤污染现状调查、污染土壤修复工作。五是实施节能减排执法行动。加快推进节能减排降碳监察执法能力建设。强化环境保护督察、环境网格化监管执法、环境风险防控。六是扎实开展生态细胞创建行动。推进文明村镇和生态村社创建活动，改善农村人居环境。加快推进幸福美丽新村建设“五大行动”，扎实开展“四好村”创建活动，建成幸福美丽新村445个、市级“四好村”499个，完成省级下达“四好村”创建目标及第三轮省市级幸福美丽新村建设示范县（片）三年目标任务的70%以上，建成核心示范片10个。

七、坚定不移统筹“七大方面”，提高人民幸福指数

加大民生投入，确保完成省上下达的民生工程、民生实事目标任务。一是强化就业创业工作。抓好高校毕业生、农村转移劳动力、城镇困难人员等重点群体就业工作，积极引导农民工返乡创业。二是进一步完善社保体系。大力推动社保经办业务下延，加快推进“三基一卡”和“社银平台”建设。三是推进保障性安居工程建设。新开工危旧房棚户区改造15747套，基本建成保障性住房和危旧房棚户区改造19426套。四是大力发展教育体育事业。普惠发展学前教育。推动义务教育均衡发展，各县（区）全面通过国家义务教育发展基本均衡县督导评估认定。加快建设宜宾体育活动中心，推动县（区）“一场一馆一池”建设，蓬勃开展全民健身活动。五是提升公共卫生服务能力。启动以全民免费体检为主要内容的全民预防保健工作，加快建立全民健康体检管理信息大数据系统。建成市残疾人康复中心。六是深入推进文化等事业发展。加快建设市博物馆、市群众文化中心，积极推进全国文明城市创建工作。扎实开展全国第三次农业普查工作。深入开展第三届全国“敬老文明号”和第五轮“四川省敬老模范县（区）”创建活动。七是创新社会治理方式。深化“平安宜宾”建设，健全立体化社会治安防控体系，严厉打击违法犯罪活动，严防暴力恐怖事件。积极开展“七五”普法和“法律十二进”，完善城乡网格化服务管理体系。严格落实安全生产责任，坚决杜绝重特大安全事故发生。强化农产品、食品药品安全监管，做好自然灾害预防和应对工作。引导群众依法表达合理诉求，促进社会和谐稳定。

八、坚定不移落实“八项举措”，优化政务服务环境

一是完善好制度。认真落实中、省已出台的制度文件，加强依法行政和运行机制建设，从职能职责、运行程序、工作要求和监督管理等方面，对政府工作规则、办文办会、督查反馈、信息调研、协调联络等制度作进一步细化和完善，提高综合行政效能。二是落实好政策。充分发挥各项财政政策和资金的作用，打好支持实体经济发展的政策措施组合拳，确保各项惠企政策落实兑现。三是拓展好市场。帮助企业搞好产销衔接、市场开拓，提升宜宾产品的市场份额。四是保障好要素。积极争取用地计划指标，清理闲置土地和盘活存量土地。做好煤、电、油、气、运等生产要素保障，确保重点行业、重点企业、重点项目的要素供给。五是搭建好平台。用好用活白酒产业发展专项资金、白酒行业互助基金，支持中小微企业发行私募债、区域绩优票据等，引导和支持有条件的企业上市融资。六是帮扶好企业。切实加强调查研究，做好经济运行的监测统计。继续实施工业企业问题清单制度和领导联系服务重点企业（项目）制度，深入开展“双千”活动，针对重点行业、重点企业、重大项目、产业园区存在的问题，逐一研究解决企业发展难题。七是提供好服务。进一步优化机关办事流程，严格落实工作责任和时限，提高服务效率、提升服务质量，全力打造优质、高效的服务环境。八是解决好落地。充分发挥监察、审计等监督作用，进一步强化督查考核，深化目标绩效管理，建立健全市委、市政府重要决策文件贯彻落实效果评价机制，着力形成齐抓共管、务实推进的良好局面。

切实加强政府自身建设

各位代表，志之所向，金石为开。面对艰巨复杂的改革发展任务，我们将不忘初心、继续前进，持续提升政府效能、改进工作作风，努力创造无愧于时代、无愧于人民的新业绩。

奋力建设法治政府。奉法者强则国强，奉法者弱则国弱。我们将切实当好尊法、学法、守法、用法的模范，始终坚持把依法行政作为政府工作的生命线，坚持依法办事、依章办事、依规办事，严格执行重大决策终身责任追究制度，做到有权必有责、失职须问责、违法必追究。我们将切实增强政治意识、大局意识、核心意识、看齐意识，自觉维护市委权威，主动接受人大工作监督和法律监督、政协民主监督以及群众监督、舆论监督，让权力在阳光下运行。

奋力建设学习政府。工欲善其事，必先利其器。我们将牢固树立终身学习的理念，坚持“博学之，审问之，慎思之，明辨之，笃行之”，以学习增长知识、增加智慧、增强本领，以学习提升决策力、提升执行力、提升创造力，努力克服本领不足、本领恐慌、本领落后等问题，不断提高工作的科学性、预见性、主动性、创造性、成效性。

奋力建设勤政政府。功崇惟志，业广惟勤。我们将始终坚持“绿色发展、科学发展、加快发展”取向，以创造全市人民更加美好的生活为奋斗目标，充分发挥集体和团队力量，在解决大事、难事和要事上心往一处想、劲往一处使，全力以赴稳增长、促改革、调结构、惠民生、防风险，以工作的成效检验执行能力、落实能力、行政能力。

奋力建设务实政府。空谈误国，实干兴邦。我们将充分发扬“钉钉子”的精神，以逢山开路、遇水搭桥的闯劲，以定了就干、干就干好的拼劲，以苦干实干、久久为功的韧劲，敢于担当、勇于负责、善作善成，全力破解落实不力、落实不到位、落实渠道不畅等难题，切实做到言必信、行必果、果必佳，进一步增强政府公信力。

奋力建设高效政府。古云此日足可珍，吾辈更应惜秒阴。我们将奉行“夙夜在公、案无留牍”的理念，当天的事当天办，坚决杜绝压件不批、压事不议、议而不决、决而慢行，全面提升行政效能，努力打造效能政府、服务政府。我们将大力整治不作为、慢作为、乱作为等庸政懒政怠政现象，切实打造愿干事、敢干事、能干成事的公务员队伍。我们将把督查督办作为倒逼落实的关键手段，以严格的督查、严厉的问责，推动全市政府系统形成比学赶超、勇争一流的良好氛围。

奋力建设廉洁政府。政者，正也。其身正，不令而行；其身不正，虽令不从。我们将严格遵守中央、省委和市委各项规定，坚持修身从严、律己从严，要求别人做到的自己首先做到，要求别人不做的自己绝对不做，切实管好亲属子女和身边的工作人员，决不以权谋私，决不拿原则做交易。严格执行招标投标相关规定，决不插手任何工程项目，决不陷入非法利益格局，以实际行动树立为民、务实、清廉的良好形象。

各位代表，立大事者，必有坚忍不拔之志。奋力夺取全市经济社会发展新胜利、开启宜宾未来新篇章，是时代的要求、组织的重托、人民的期盼，也是政府必须肩负的神圣使命。让我们更加紧密地团结在以习近平同志为核心的党中央周围，在省委、省政府和市委的坚强领导下，在市人大及其常委会和市政协的监督支持下，紧紧依靠全市各族人民，凝心聚力、砥砺奋进，实干争先、富民强市，为决战决胜全面小康而努力奋斗！
